# Design, development, and evaluation of a local sensor-based gait phase recognition system using a logistic model decision tree for orthosis-control

**DOI:** 10.1186/s12984-019-0486-z

**Published:** 2019-02-01

**Authors:** Johnny D. Farah, Natalie Baddour, Edward D. Lemaire

**Affiliations:** 1 The Ottawa Hospital Research Institute, Ottawa-Carleton Institute of Biomedical Engineering, Ottawa, ON K1N 6N5 Canada; 2 Department of Mechanical Engineering, University of Ottawa, Ottawa, ON K1N 6N5 Canada; 3 Ottawa Hospital Research Institute, Ottawa, ON K1H8M2 Canada; 4 University of Ottawa Faculty of Medicine, Ottawa, ON K1H 8M5 Canada

**Keywords:** Knee ankle foot orthosis, Stance control, Machine learning, Decision tree, Gait phase recognition, Sensors, Microprocessor

## Abstract

**Background:**

Functionality and versatility of microprocessor-controlled stance-control knee-ankle-foot orthoses (M-SCKAFO) are dictated by their embedded control systems. Proper gait phase recognition (GPR) is required to enable these devices to provide sufficient knee-control at the appropriate time, thereby reducing the incidence of knee-collapse and fall events. Ideally, the M-SCKAFO sensor system would be local to the thigh and knee, to facilitate innovative orthosis designs that allow more flexibility for ankle joint selection and other orthosis components. We hypothesized that machine learning with local sensor signals from the thigh and knee could effectively distinguish gait phases across different walking conditions (i.e., surface levels, walking speeds) and that performance would improve with gait phase transition criteria (i.e., current states depend on previous states).

**Methods:**

A logistic model decision tree (LMT) classifier was trained and tested (five-fold cross-validation) on gait data that included knee flexion angle, thigh-segment angular velocity, and thigh-segment acceleration. Twenty features were extracted from 0.1 s sliding windows for 30 able-bodied participants that walked on different surfaces (level, down-slope, up-slope, right cross-slope, left cross-slope) at a various walking speeds (self-paced (1.33 m/s, SD = 0.04 m/s), 0.8, 0.6, 0.4 m/s). The LMT-based GPR model was also tested with another validation set containing similar features and surfaces from 12 able-bodied volunteers at self-paced walking speeds (1.41 m/s, SD = 0.34 m/s). A “Transition Sequence Verification and Correction” (TSVC) algorithm was applied to correct for continuous class prediction and to improve GPR performance.

**Results:**

The LMT had a tree size of 1643 with 822 leaf nodes, with a logistic regression model at each leaf node. The local sensor LMT-based GPR model identified loading response, push-off, swing, and terminal swing phases with overall classification accuracy of 98.38 for the initial training set (five-fold cross-validation) and 90.60% for the validation set. Applying TSVC increased classification accuracy to 98.72% for the initial training set and 98.61% for the validation set. Sensitivity, specificity, precision, F-score, and Matthew’s correlation coefficient results suggest strong evidence for the feasibility of an LMT-based GPR system for real-time orthosis control.

**Conclusions:**

The novel machine learning GPR model that used sensor features local to the thigh and knee was viable for dynamic knee-ankle-foot orthosis-control. This highly accurate GPR model was generalizable when combined with TSVC. Our approach could reduce sensor system complexity as compared with other M-SCKAFO approaches, thereby enabling customizable advantages for end-users through modular unit orthosis designs.

## Introduction

Stance-Control Knee-Ankle-Foot Orthoses (SCKAFO) are wearable walk-assist devices that prevent the knee from collapsing during weight-bearing and provide unhindered knee motion during swing. SCKAFO can improve mobility and provide more natural gait than conventional fixed-knee KAFOs for people with knee-extensor pathologies [1–5]. Fewer compensatory gait mechanisms (i.e., knee hyperextension, hip-hiking, circumduction, vaulting [1]) can reduce associated joint loads, reduce energy consumption [6, 7], increase foot clearance, increase walking speeds, and improve overall user satisfaction [1, 2, 4–6].

SCKAFO mechanical knee joint designs have their own advantages and disadvantages [4, 5]. Some designs use weighted/spring-loaded pawls or belt clamping [1, 2, 4] to lock the knee at initial contact and disengage at foot-off, based on leg position. Most mechanically-controlled SCKAFO require full knee extension to engage knee-lock [4, 5, 8]. This can present difficulties for individuals who cannot extend their leg at each step, making continuous and reliable stance-control more difficult. Persons with knee-extensor weakness that have sufficient hip-flexion control could also use angular velocity based stance-control [3, 8, 9], where mechanical components at the knee engage knee-flexion resistance at any knee angle once an angular velocity threshold is passed, such as during a knee collapse or fall event (i.e., body weight sensing not required).

The main advantages of mechanically-controlled SCKAFO include free knee motion during swing, simpler stance-control systems that do not require external power sources, low profile, lightweight, and ability to fit under trousers. Unfortunately, mechanically-controlled SCKAFO can have inconsistent stance/swing phase recognition, leading to unreliable locking and unlocking and poor functional versatility across different walking conditions. Mechanically-controlled SCKAFO can also have difficulty negotiating between different walking speeds, gait modes, terrains, and daily living environments (i.e., stairs, ramps, uneven ground) [3–5, 8, 10–12].

Microprocessor-controlled SCKAFO (M-SCKAFO) guide knee control by regulating between stance and swing using multiple electronic sensors attached to various orthotic-limb segments [4–6, 9, 10, 12–20]. These electronic sensors, coupled with computational algorithms, dictate when to engage/disengage knee-flexion resistance. This provides enhanced knee-control functionality, reliability, and versatility across surfaces and walking speeds. M-SCKAFO may also toggle between different gait modes (i.e., ramps, curbs, stairs) [4, 5, 12]. These devices rely on many sensors and complex algorithms that can have high computational costs and require external power sources that require regular charging. M-SCKAFO drawbacks include sensors located at many orthosis segments, bulk (i.e., cannot fit under trousers), lack of aesthetics, high cost, and fewer orthotic component choices.

The Otto Bock C-Brace [21] is currently the most versatile commercially available M-SCKAFO, using a hydraulic linear damper for knee control. C-Brace is the only M-SCKAFO on the market with in-stance knee flexion dampening (i.e., gradually allows the knee to flex during stance). With partial knee-flexion resistances, some individuals with lower limb muscle impairment could re-establish sufficient strength and mobility to eliminate the need for ambulatory assistance. The C-Brace uses two sets of sensors at the knee to determine knee angle and a dorsal shank spring (strain-gauge ankle-moment sensor) to determine when the person is weight-bearing [5].

Electronic sensors, such as inertial measurement units (IMU), enable signal-processing algorithms to determine limb orientation and/or position in the gait cycle and prompt the knee joint mechanism to switch to a locked, free, or partial knee flexion-resistance setting. Ideally, an effective stance-control system would provide support during stance and unhampered knee motion during swing, for natural gait. Knee stability is of paramount importance for safe gait, but does not always require knee extensor contributions [22]. During stance, knee motion is predominantly in extension. However, the onset of knee flexion occurs between terminal stance and pre-swing. The body has forward impetus during this phase, with controlled ankle and hip removing the need for knee extensor control [22]. Safe knee-release must occur without active quadriceps muscle activation, prior to knee-flexion during swing, and after the contralateral limb is in contact with the floor. Moreover, stance-control systems must identify loading response to securely lock and keep the knee from collapsing. These criteria provide knee-release transition points that ensure safe-gait by not imposing unmanageable loads on weak knee-extensors. Consequently, accurate gait phase recognition (GPR) from wearable sensor data becomes essential for real-time orthosis-control.

Emerging gait analysis techniques use embedded sensors in wearables and offer practical modalities for gait monitoring, human activity recognition (HAR), and prosthesis and orthosis control. Electronic sensors such as strain gauges [21], pressure sensors [23], electromyography (EMG) [17], force sensitive resistors [24–26], goniometers [27, 28], and IMU [10, 12, 23–26, 29–34] can be combined to give highly accurate and real-time gait monitoring.

Liu, et al. [35] placed a two-axis accelerometer and three gyroscopes on the foot, shank and thigh to detect gait phases (initial contact, loading response, mid-stance, terminal stance, pre-swing, initial swing, mid-swing, terminal swing) and provide limb segment orientation. Pappas et al. [25] reported a rule-based gait phase identification system across able-bodied and impaired individuals, with states identified by prior characterization of IMU signals. Their system used foot angular velocity and three force sensitive resistors to determine weight-bearing. To improve GPR performance, they implemented knowledge-based gait phase transitions to determine possible changes of state during the gait cycle. Gorsic et al. [23] developed a gait phase identification system to provide feedback for a lower-limb robotic prosthesis. The system used IMU attached to body segments and shoe insole sensors to determine four gait phases (left stance, left-right double stance, right stance, right-left double stance). Another study [36] attempted to localize a GPR sensor system using a single IMU in a trouser pocket. They did not implement a computer-based algorithm to determine gait phases but showed that major gait phases could be visually identified from the thigh-angle signature. This is important for lower limb orthosis-control since many M-SCKAFO can benefit from computer-based/real-time GPR, along with modular electronic components located only at the thigh [10, 12].

Machine learning and artificial intelligence approaches related to HAR are becoming increasingly popular [31, 37–40]. Machine learning classifiers can provide robust, fast, and accurate classification from simple features extracted from biomechanical data, making them highly attractive for use in GPR [10, 23, 24, 41]. Machine learning algorithms coupled with local sensor systems have demonstrated sufficiently good performance to make this a potentially viable approach for identifying current states and recognizing human intent for control system feedback.

The objective of this research is to propose a method for identifying walking phases with only local gait data from the thigh and knee. Accurate GPR would provide essential information for M-SCKAFO control if the approach works in real-time, across different surface-levels, and across walking speeds. We hypothesized that signal features from the thigh and knee with a logistic decision tree machine learning model could provide highly accurate GPR performance. Secondly, a “Transition Sequence Verification and Correction” (TSVC) algorithm should improve classification results. The model should classify walking phases regardless of surface-level, walking speed, or individual walking variability. Appropriate GPR with sensors about the knee will enable new M-SCKAFO approaches that allow orthotists to use the most appropriate ankle and foot designs for the user, without limitations due to ankle-foot sensor system requirements in current devices.

## Methods

### Data set

A de-identified data set from 30 able-bodied participants was used in this study (Ottawa Health Science Network Research Ethics Board approval (20140825-01H). Walking data were collected in a Computer-Assisted Rehabilitation Environment (CAREN-Extended) (Motek Medical, Amsterdam, NL) virtual environment system. CAREN-Extended consists of a six degree-of-freedom force-plate platform with 1 m × 2 m dual tread instrumented treadmill (Bertec Corp., Columbus, OH), 180° screen for virtual world projection, and a 12-camera 3D motion capture system (Vicon Inc., Oxford, UK). A full body marker set defined all joint and body-segment positions [42, 43]. Marker data were recorded at 100 Hz and ground reaction forces were recorded at 1000 Hz.

Each volunteer walked in a custom-built virtual park application on level (LG), 7^°^ declination down-slope (DS), 7^°^ inclination up-slope (US), 5^°^ inclination right cross-slope (RS), and 5^°^ inclination left cross-slope (LS), at self-paced (SP) speed (1.33 m/s, SD = 0.04 m/s), 0.8 m/s, 0.6 m/s, and 0.4 m/s. Joint and body-segment trajectories were imported into Visual 3D (C-Motion Inc., Germantown, MD) for biomechanical analyses. A fourth order low pass Butterworth filter with 10 Hz cut-off frequency was applied to the marker data before calculating joint and segment kinematics.

Ten strides for knee flexion-extension angle (KA), thigh-segment angular velocity in each axis (AngVel), and thigh-segment acceleration in each axis (Acc) were extracted from each walking condition (surface and speed) and imported into Matlab. All data were linearly interpolated to 200 Hz to mimic an IMU internal sampling rate for M-SCKAFO control systems.

### Validation set

A separate de-identified data set from 12 able-bodied participants provided analogous gait data to the training data set, using the same testing protocol. Six strides from each surface condition were extracted for SP walking (1.41 m/s, SD = 0.34 m/s). This set can be considered as unseen data.

### Gait phase recognition

Gait phase segmentation was implemented using a custom Matlab program with predefined gait events that directly corresponded to gait signatures used. KA, AngVel (*x, y, z*), and Acc (*x, y, z*) signals were partitioned into four gait phases: Loading Response (LR), Push-Off (PO), Swing, and Terminal Swing (TSw) according to initial contact, mid-stance, foot-off, and maximum knee flexion angle gait events [10, 12]. AngVel and Acc resultants were used as supplementary signals from which features were extracted for machine learning implementation. Equation (1) describes the resultant signal for AngVel and Acc.

1 $$ \sqrt{a_x^2+{a}_y^2+{a}_z^2,} $$

where *a*_*x*_*, a*_*y*_, and *a*_*z*_ are the AngVel and Acc signals from *x, y, z* orientations, respectively.

Correlation-based feature selection was applied in a preliminary analysis [12], providing 20 features: KA mean (*x*-axis), AngVel mean (*x*-axis, *y*-axis), Acc mean (y-axis, z-axis), KA variance (*x*-axis), KA maximum difference (*x*-axis), AngVel maximum difference (*x*-axis), KA minimum (*x*-axis), AngVel minimum (*y*-axis), Acc maximum (*y*-axis), AngVel sign-sum (*x*-axis, *y*-axis, *z*-axis), Acc sign-sum (*x*-axis, *y*-axis, *z*-axis), resultant Acc sum of peaks (total number of peaks), AngVel principal frequency (the frequency component with the greatest magnitude with a Fast Fourier Transform (FFT), *y*-axis), and the correlation coefficient between the *z*-axis (parallel to gravity during stance) and *y*-axis (parallel to heading during stance and swing) acceleration. The sign-sum was defined as the sum of scores for each data point in a sliding window (negative values were scored as − 1, and positive values were scored as + 1). All features were extracted from a 0.1-s sliding window with 90% overlap (incremented at 0.01 s) and labelled as LR, PO, swing, or TSw according to the last data point [10, 12] in each window.

#### Logistic model decision tree

The feature vector was imported into the Waikato Environment for Knowledge Analysis (WEKA) for machine learning implementation. A logistic model decision tree (LMT) [44] classifier was trained and tested with 5-Fold Cross Validation (5-FCV) using stratified data splits. Performance metrics were obtained by reiterating through the supplied training set. The LMT was constructed as a J-48 Decision Tree with logistic regression models at terminal leaf nodes. Node splitting involved the C4.5 decision tree splitting criterion [44–46], using information gain on the class variable. Logistic regression functions at leaf nodes were determined using LogitBoost heuristic [44]. Default LMT parameters were used to construct the tree (i.e., convertNominal = False, debug = False, errorOnProbabilities = False, fastRegression = True, minNumInstances = 15, numBoostingIterations = − 1, splitOnResiduals = False, useAIC = False, weightTrimBeta = 0).

The LMT was manually implemented into a Matlab script as a function. The function took instances (vector containing features computed from sliding window signature) as input and returned the gait phase corresponding to the LMT model with the greatest probability as the gait phase prediction. The logistic function, shown by Eq. (2) is a sigmoid function and computes the probability of being in a gait phase.

2 $$ P\left({\gamma}_i\right)=\frac{e^{\gamma_i}}{1+{e}^{\gamma_i}} $$

where *γ*_*i*_ contains the coefficients of determination that form the linear combination of multiple variables (i.e., features) for logistic regression and correspond to the gait phase, *i*.

#### Transition sequence verification and correction

A “transition sequence verification and correction” (TSVC) algorithm was applied after the GPR function class output to resolve discontinuous class complications. The continuous sequence was defined as LR, PO, Swing, TSw, and repeated for the following stride. TSVC checks if the current instance is different than the three previous instances. If different and not the next phase in the sequence, the instance would be relabeled as the previous instance (e.g., “LR, LR, LR, **Swing**” relabelled as “LR, LR, LR, **LR**”). Additionally, if an outlier classification occurred between four consecutive instances, the set of three previous and a single next instance are evaluated for an outlier classification where the outlier would be relabelled as the previous instance (e.g., “LR, LR, LR, **PO**, LR” relabelled as “LR, LR, LR, **LR**, LR”). This GPR model and TSVC process also indirectly identified gait events (e.g., transition between TSw and LR indicates initial contact).

**Fig. 1:**
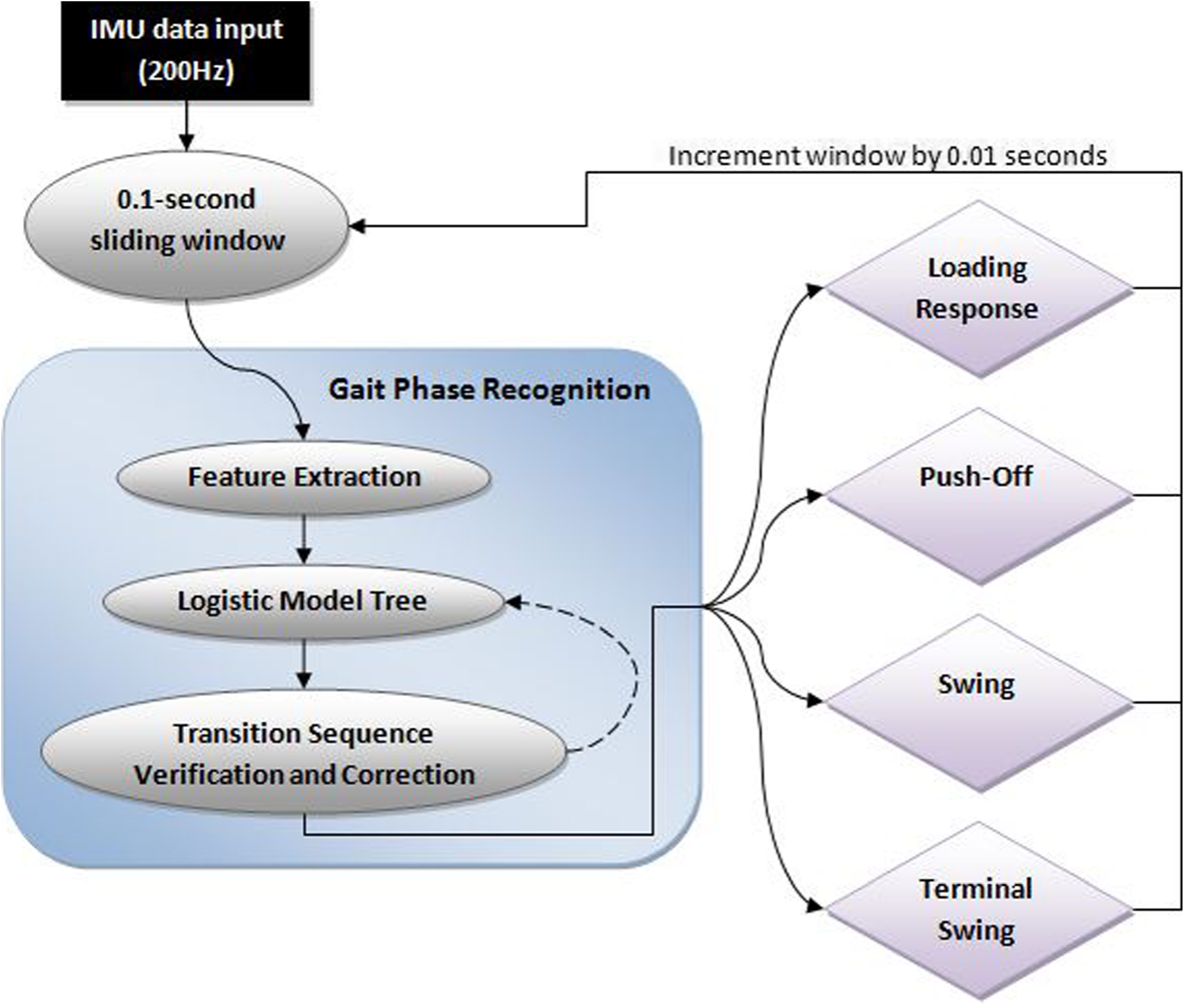
Gait phase recognition flow diagram

As shown in Fig. 1, sensor input from KA, AngVel, and Acc were stored in a 0.1 s sliding window (20 data points, 200 Hz sampling rate) for feature extraction and LMT-based GPR would be performed. TSVC verified and corrected for continuous gait phase sequence and output a classification. The system then incremented the sliding window by 0.01 s to classify the next instance.

#### Classifier evaluation

Classification performance metrics included tree size, number of leaf nodes, overall classification accuracy, sensitivity (Sens), specificity (Spec), precision (Prec), F-score (FS), Matthews Correlation Coefficient (MCC). Classification metrics were computed from true positives (TP), true negatives (TN), false positives (FP), and false negatives (FN) using WEKA by supplying the feature matrix as the test set. Classification metrics for LMT with TSVC were computed from a confusion matrix implemented in Matlab2016b, with weighted averages given by

3 $$ {W}_m=\frac{\sum_i{m_i}^{\ast }{I}_i}{\sum_i{I}_i}, $$

where *m* denotes a classification metric, *i* denotes a specific gait phase, *I* denotes the total number of instances in the total feature matrix relating to each gait phase.

## Results

### Gait phase recognition

**Fig. 2:**
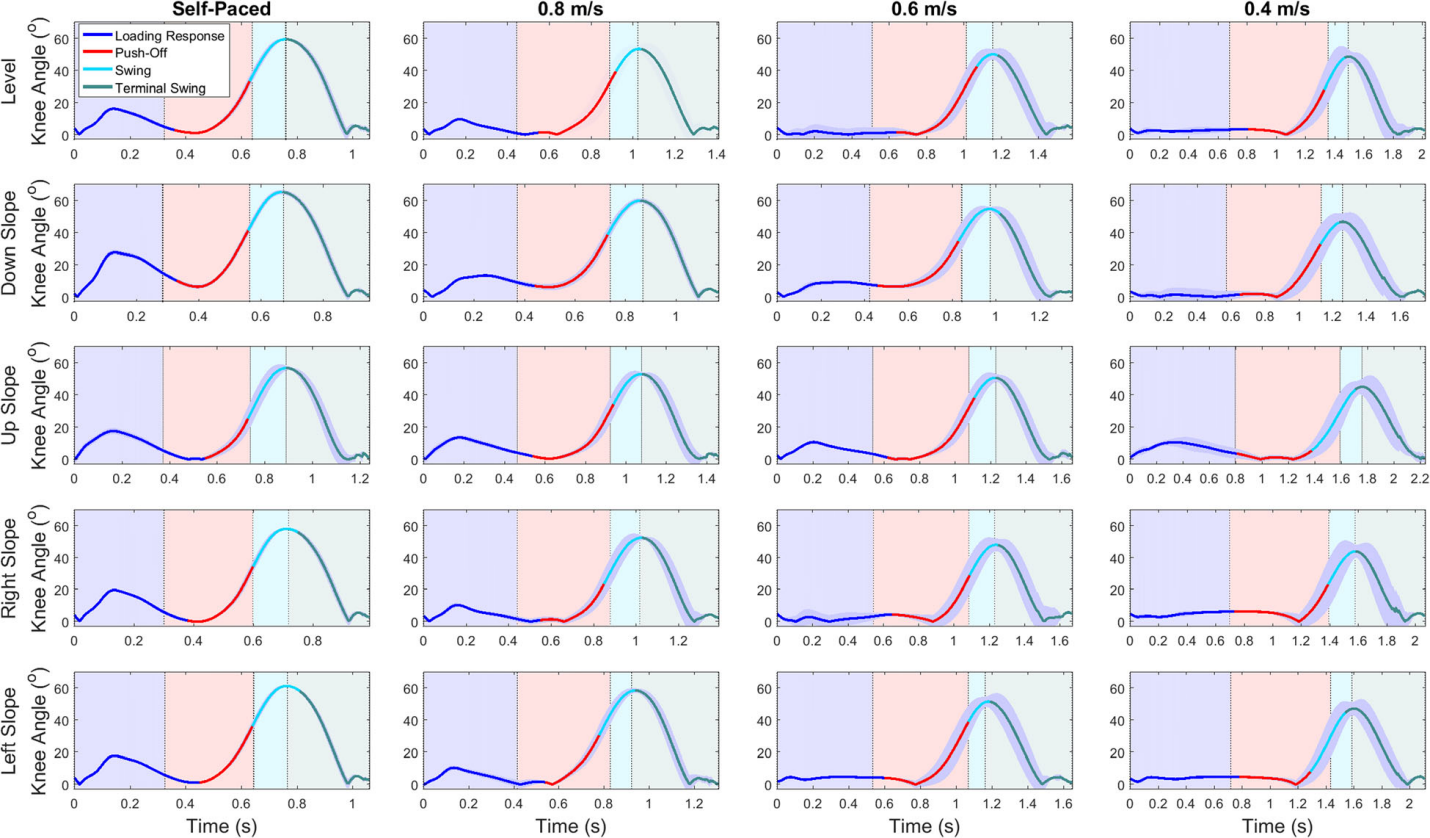
Average knee angle (degrees) and standard deviation from a single participant for each surface condition (rows) and walking speed (columns), showing classified gait phases: Loading Response (blue), Push-Off (red), Swing (cyan), Terminal Swing (teal) for the training set. Shaded areas behind the signatures show true classes along the stride and vertical lines represent gait events

**Fig. 3:**
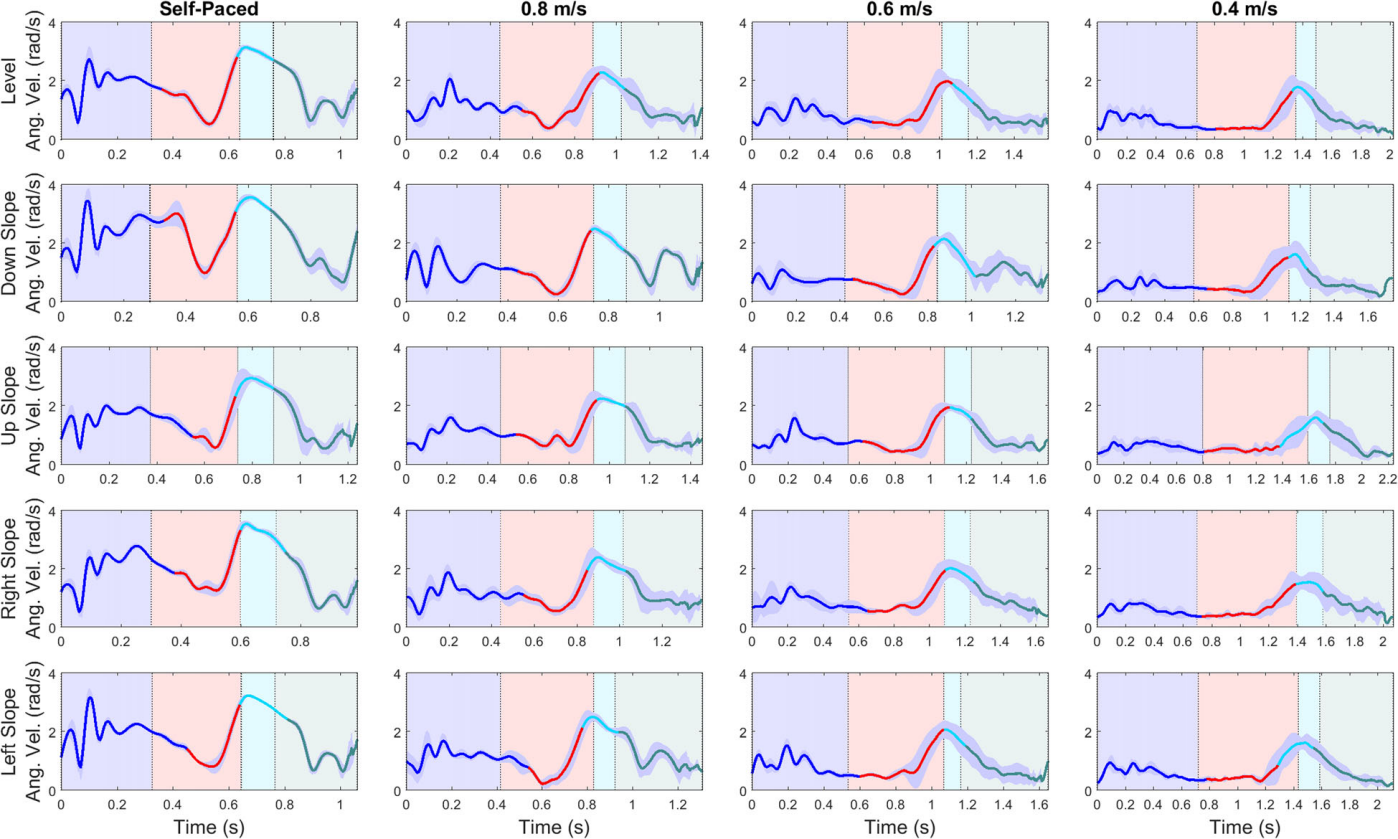
Average thigh-segment angular velocity (rad/s) and standard deviation (shaded) from a single participant for each surface condition (rows) and walking speed (columns), showing classified gait phases: Loading Response (blue), Push-Off (red), Swing (cyan), Terminal Swing (teal) for the training set. Shaded areas behind the signatures show true classes along the stride and vertical lines represent gait events

**Fig. 4:**
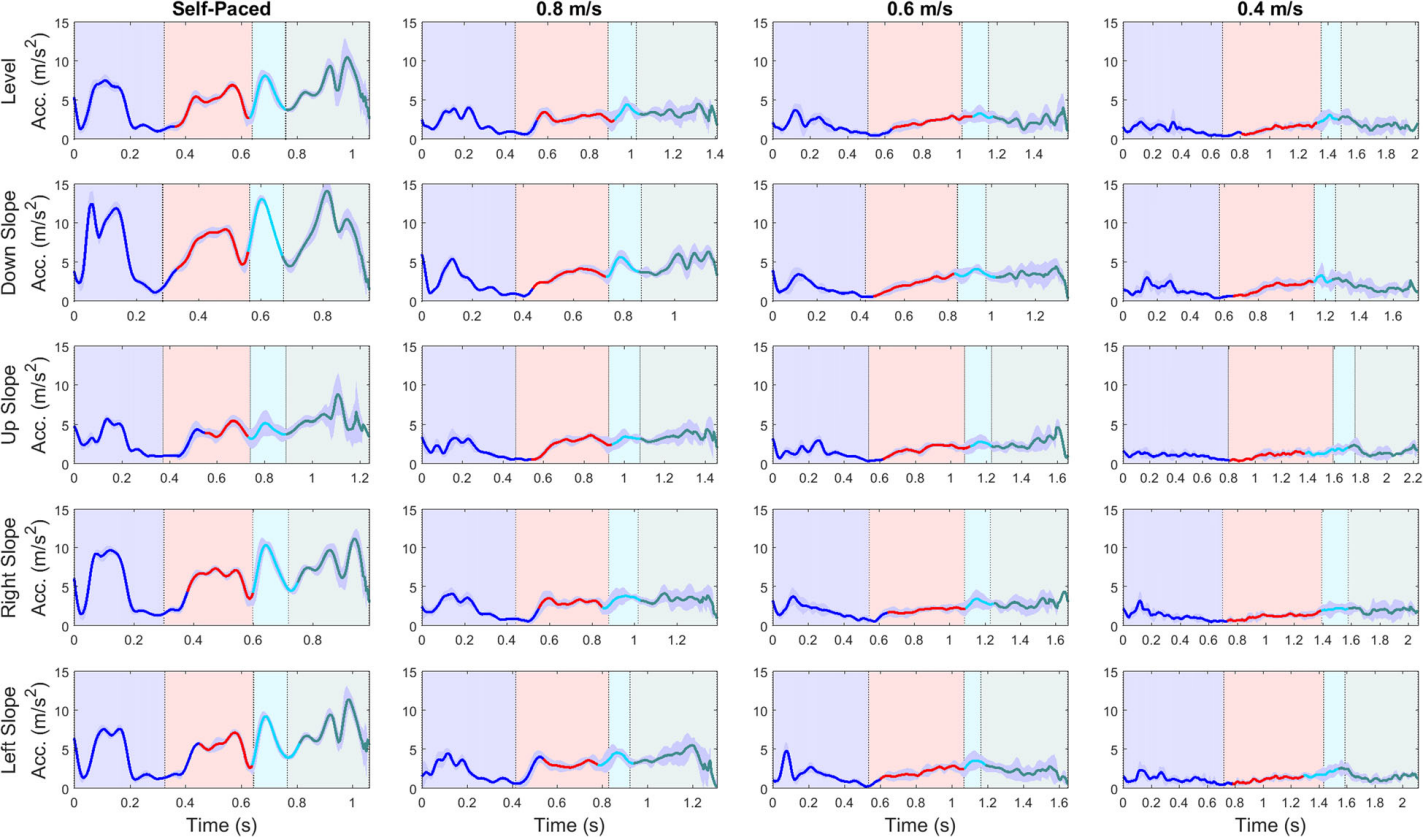
Average thigh-segment acceleration (m/s^**2**^) from a single participant for each surface condition (rows) and walking speed (columns), showing classified gait phases; Loading Response (blue), Push-Off (red), Swing (cyan), Terminal Swing (teal) for the training set. Shaded areas behind the signatures show true classes along the stride and vertical lines represent gait events

The LMT size was 1643 with 822 leaf nodes, with a logistic regression function at each leaf node. Predicted gait phases for KA (Fig. 2), resultant AngVel (Fig. 3), and resultant Acc (Fig. 4) show partitioned gait phases during strides with LMT classification and coincide well with true classes (shaded areas). Vertical lines shown between shaded areas represent gait events used for stride segmentation. Applying LMT classification indicates that our GPR model can indirectly detect those events across walking conditions. Similar curve shapes are observed across different surfaces and signal variation increases for slower walking speeds. Fig. 2 shows that for slower speeds the stance phase (i.e., LR, PO), knee motion decreases. However, the GPR model was able to distinguish all gait phases regardless of surface condition and walking speed.

Table 1 presents the confusion matrix for the training set and Table 2 presents the confusion matrix for the validation set. Shaded boxes represent TP classifications where the GPR model successfully identified gait phases, whereas off-diagonal boxes indicate a misclassification.

**Table 1:** Training Set Confusion Matrix

True Class	Classified As			
	Loading Response	Push-Off	Swing	Terminal Swing
Loading Response	240,409	3972	0	178
Push-Off	4884	290,454	2008	1
Swing	1	1192	76,349	696
Terminal Swing	266	0	578	227,101

**Table 2:** Validation set confusion matrix

True Class	Classified As			
	Loading Response	Push-Off	Swing	Terminal Swing
Loading Response	16,433	841	12	477
Push-Off	2679	19,073	629	1272
Swing	1	323	8309	56
Terminal Swing	0	74	141	19,288

Table 3 presents the LMT classification metrics for training set (30 able-bodied participants) and validation set (12 able-bodied participants). Weighted averages are with and without TSVC.

**Table 3:** Logistic model tree classification performance

Model	Data Set	Accuracy	Sensitivity	Specificity	Precision	F-score	MCC
LMT	Training	98.38	0.98	0.99	0.98	0.98	0.98
	Validation	90.60	0.91	0.97	0.91	0.91	0.87
LMT + TSVC	Training	98.76	0.97	0.99	0.97	0.97	0.96
	Validation	98.61	0.97	0.99	0.97	0.97	0.96

Gait phase classification performed well for the training set, with 98.38% overall accuracy and all classification metrics greater than 0.98. TSVC increased accuracy by 0.38%; sensitivity, and FS decreased by 1%; MCC decreased by 2%; and specificity remained the same.

Validation set metrics were less than the training set, with lower accuracy (− 7.78%), sensitivity (− 7%), specificity (− 2%), precision (− 7%), FS (− 7%), and MCC (− 11%). However, after applying TSVC, all validation set classification metrics improved. Accuracy increased by 8.01%, sensitivity, precision and FS increased by 6%, specificity increased by 2%, and MCC increased by 9%, with all results greater than 0.96.

Table 4 shows classification metrics for each gait phase. All classification metrics were greater than or equal to 0.97 across all gait phases. TSw performed the best with all classification metrics at 0.99. Swing had the lowest precision and FS. For the validation set, excluding PO, classification metrics were all greater than 0.85. PO had the lowest sensitivity, FS, MCC. LR had the second lowest sensitivity and the lowest specificity. In terms of classification performance metrics, Swing and TSw performed similarly and outperformed LR and PO for unseen data.

**Table 4:** LMT-GPR performance by gait phase

Data Set	Gait Phase	Sensitivity	Specificity	Precision	F-score	MCC
Training	Loading Response	0.98	0.99	0.98	0.98	0.97
	Push-Off	0.98	0.99	0.98	0.98	0.97
	Swing	0.98	0.99	0.97	0.97	0.97
	Terminal Swing	0.99	0.99	0.99	0.99	0.99
Validation	Loading Response	0.91	0.95	0.87	0.89	0.85
	Push-Off	0.82	0.97	0.93	0.87	0.81
	Swing	0.96	0.99	0.92	0.94	0.93
	Terminal Swing	0.99	0.96	0.91	0.95	0.93

Table 5 shows performance metrics for the LMT with TSCV, for specific gait phases. All results were greater than 0.91, for the training set. Compared to results in Table 4, sensitivity decreased for PO and Swing, and remained the same for LR and TSw. Swing had the lowest sensitivity, FS, and MCC. TSw had the best performance across all classification metrics equal to 0.99, apart from MCC. However, MCC was still greater for the TSw phase than MCC for all other gait phases.

**Table 5:** LMT + TSVC GPR performance by gait phase

Data Set	Gait Phase	Sensitivity	Specificity	Precision	F-score	MCC
Training	Loading Response	0.98	0.99	0.97	0.98	0.97
	Push-Off	0.96	0.99	0.94	0.95	0.94
	Swing	0.91	0.99	0.97	0.94	0.93
	Terminal Swing	0.99	0.99	0.99	0.99	0.98
Validation	Loading Response	0.99	0.99	0.99	0.99	0.99
	Push-Off	0.98	0.98	0.90	0.94	0.93
	Swing	0.87	0.99	0.95	0.91	0.90
	Terminal Swing	0.97	0.99	0.99	0.98	0.98

For the validation set, all but one metric (0.87) was greater than 0.90. Compared to the training set, all classification metrics increased for LR and TSw. All metrics increased for PO for the validation set apart from precision (decreased by 8%). Swing decreased for sensitivity (9%), FS (3%), MCC (3%). LR classification performance for the validation set outperformed all other gait phases.

## Discussion

This research demonstrated the viability of a local sensor, machine learning-based, gait phase recognition system to guide decision-making for orthosis-control and safe mobility. The LMT machine learning classifier successfully performed GPR for small data windows, enabling real-time device control across different surfaces and walking speeds encountered throughout daily living activities.

### Gait phase recognition classifier design and development

The machine-learning model reliably recognized LR, PO, Swing, and TSw, defined by gait events [10], across LG, DS, US, RS, and LS at different walking speeds. In the literature, most GPR models were trained and tested on level-ground walking at self-paced speeds [10, 24, 26, 30, 41], with few studies testing on a variety of surfaces [12, 25]. Models trained with multiple walking conditions that occur throughout daily living activities could enhance generalizability [15] and overall system functionality across daily living activities.

Since our GPR model was trained from a data set that contained simple data features extracted from small data windows (overlapping 0.1 s sliding window) from local thigh and knee signals across a variety of walking conditions, results can be directly translated to real-time lower-limb orthosis control. These features are not computationally intensive, facilitating fast GPR decision-making throughout the gait cycle on embedded joint electronics.

In a preliminary analysis [10, 12], a J-48 DT was chosen as the machine learning classifier due to success in activity recognition and gait applications [25, 29, 38–40], exceptional gait phase classification performance [10, 12], and ability to work in real-time. In this study, the classifier implemented logistic regression models at each terminal node to handle multi-class target variables. This provided gait phase probability estimates rather than just a classification output, and when correctly pruned produced a smaller tree than ordinary classification trees [44]. The J-48 DT previously used for local sensor GPR was very large, with a tree size of 16,103 and 8052 leaf nodes [12]. Generally, a smaller decision tree is a simpler model and is expected to provide computational efficiency in terms of battery consumption and classification time on a M-SCKAFO onboard microprocessor. The machine learning classifier constructed in this study, with the same training data and walking conditions, produced a tree that was approximately 90% smaller and had 90% fewer terminal leaf nodes than J-48 DT [12]. For GPR in real-time orthosis control, high accuracy and generalizability becomes more important than tree size, as long as classification decisions can be made within an appropriate period.

Classification performance also improved for all metrics. These results are in agreement with Landwehr, et al. [44] where LMT outperformed C4.5 DT (similar to J-48 DT) on 14 different data sets and LMT size was significantly less than C4.5 DT size for 16 data sets. For our study, improved gait phase classification was likely due to the probability estimates from logistic regression functions at each node. Branches in the tree would guide input information towards a node based on our highly representative training set (i.e., 30 able-bodied participants, surface levels, walking speeds), and introduced an additional probabilistic measure (i.e., logistic regression at terminal nodes) to determine class output. The LMT model’s structure and characteristics enable easier, faster, and more accurate GPR implementation on a microprocessor simply due to the model’s size and therefore computational complexity. In addition, a simpler model is more practical for adding deeper, supplementary rule-based algorithms (i.e., sequence transitions, knee-release, stumble control) thereby improving assistive-device designs, and machine learning-based stance-control feasibility.

Results from 5-FCV on the training set were very good, across all gait phases. MCC values suggest that, regardless of class imbalances within our data set, PO and Swing classifications performed well with TSVC. Sensitivity was less than specificity in all cases. High sensitivity and specificity are essential since specificity errors could result in the system switching to a knee-release setting during weight-bearing (e.g., mistaking LR for Swing), putting the user at risk of falling. Similarly, sensitivity errors could result in the joint failing to switch to knee-release when transitioning from PO to Swing. In practice, maintaining knee support during weight bearing is a priority for fall-prevention. Therefore, high specificity is advantageous for stance-control applications.

Overall classification accuracy improved with TSVC algorithm implementation, but some evaluation metrics diminished slightly. Interestingly, implementing the model on the full training set with the TSVC algorithm improved overall classification accuracy. The decrease in sensitivity for PO and Swing meant that TP were missed (i.e. FN), likely due to forcing a transition if a particular class was mistaken for any another class. Swing phase was expected to occur after PO and had fewer data instances; hence the greater decrease in sensitivity for Swing. During the transition from PO to Swing, Swing onset could be classified as PO for slower walking speeds where the signals appear similar (Figs. 2, 3 and 4). Stance phase increases for slower speeds to increase double support time and maintain stability [47]. Certain implications and difficulties arise when implementing stance-control at extremely slow walking speeds, such as 0.4 m/s. This is mainly due to the lack of signal variation at these locations during the stride, as the mean standard deviations (MSD) are close to zero. Ideally, with a sufficient static versus dynamic state decision algorithm [48], stance-control could be applied with a local sensor system at very slow walking speeds. Literature regarding GPR for very slow walking is sparse. Multiple GPR models trained to particular walking speeds (i.e., walking speeds ≤0.4 m/s) may be better to determine appropriate knee flexion resistance engagement/disengagement. Since this would require an algorithm for determining walking speed, which is difficult to accurately determine from IMU data, surrogate measures such as cadence or step time could be considered.

The GPR model had promising classification performances and demonstrated that our model could be a viable solution in localized intelligent sensor systems for M-SCKAFO applications. Based on overall classification accuracy, both of hypotheses are valid (i.e., sensor signal features from the thigh and knee with a LMT model could provide highly accurate GPR performance; a TSVC algorithm should improve classification results). However, given that decision trees are low-bias classifiers, they are expected to capture regularities in the training data. Hence, the GPR model was also evaluated with a different data (validation set).

### Validation set evaluation

As expected, LMT classifier results were less with the validation set (12 different participants) than the training set. While overall classification accuracy was still greater than 90% for all evaluation metrics, a 10% error rate is not appropriate for assistive device-control, indicating that our initial hypothesis was not valid when the model was applied generally. The TSVC algorithm improved classification accuracy with the validation set since gait phases are sequential when walking. This suggests that transition correction algorithms may improve GPR models and validates the second hypothesis for new gait data.

Knowing where and when misclassifications occur is important for knee engagement/disengagement in M-SCKAFO implementation. FN occurred during gait phase transitions, where gait signals exit one phase and enter the next. For small data windows (0.1 s), feature values could be similar for consecutive windows with 90% overlap. Therefore, at regions where one gait phase transitions into another, class distributions will not vary without compelling gradient-changes in the signal. For example, if misclassifications are present at the beginning of swing, these instances would be classified as PO, thereby engaging knee-release. However, misclassifying instances near the middle or end of Swing may engage knee-flexion-resistance too early and perturb free knee-motion. As shown by Fig. 2 (KA), Fig. 3 (AngVel), and Fig. 4 (Acc) gait phase transitions are late for LR to PO. For the LMT tested with the validation set and TSVC, results showed that 1.8% of instances were classified as PO but were actually Swing and 0.5% of instances were classified as Swing but were actually TSw (i.e., misclassification at the beginning of swing, rather than at the end). Misclassifications at the beginning of Swing are shown in Figs. 2, 3 and 4 for US and LS surfaces at 0.4 m/s walking speed. Since this study involved analysis on right limb data only, right limb range of motions are similar for US and LS surfaces. This is important for misclassified instances at the beginning of swing for initiating knee-engagement or knee-disengagement. For an M-SCKAFO, knee-release would ideally occur during PO, at the earliest safe knee-release point (i.e., onset of knee flexion during in pre-swing, when weight-bearing is declining). Custom M-SCKAFO control adjustments for the participant could help achieve this ideal PO-swing transition point.

In summary, GPR model classification performance with the validation set improved with TSVC and was comparable to training set results.

### Limitations

The Vicon motion analysis system provided very accurate thigh and knee data. Depending on the sensors used in the orthotic application (i.e., IMU, etc.), data in practice may be of lower quality, or have additional noise and errors due to the environment. Appropriate sensor technologies are needed to ensure translation of the research outcomes.

## Conclusion

A logistic decision tree gait phase recognition model successfully identified loading response, push-off, swing, and terminal swing gait phases for five different surfaces and a range of walking speeds, with input data localized to the thigh and knee. The logistic model decision tree classifier was robust for many simulated walking conditions experienced throughout daily living and was generalizable to unseen data from a different participant group. A gait phase transition sequence verification and correction algorithm was essential to achieve appropriate GPR performance results. This research provides supporting evidence that machine learning can provide enhanced gait phase recognition for real-time orthosis-control across multiple real-world walking scenarios. Local sensors at the thigh and knee reduce sensor system complexity and help to provide an integrated, modular unit for microprocessor-controlled stance-control knee-ankle-foot orthoses.

## Abbreviations

5-FCV: 5-Fold Cross Validation
Acc: Thigh-Segment Acceleration
AngVel: Thigh-Segment Angular Velocity
CAGH: Correlation Along Gravity (z-axis acceleration) and Heading (y-axis acceleration)
CAREN-Extended: Computer Assisted Rehabilitation Environment
CFS: Correlation-Based feature selection
DS: Down Slope
FN: False Negative
FP: False Positive
FS: F-Score
GPR: Gait Phase Recognition
HAR: Human Activity Recognition
IMU: Inertial Measurement Unit
J-48 DT: J-48 Decision Tree
KA: Knee Angle
KAFO: Knee-Ankle-Foot Orthoses
LG: Level Ground
LG-DS-US: Level Ground-Down Slope-Up Slope
LG-RS-LS: Level Ground-Right Slope-Left Slope
LMT: Logistic Model Tree
LR: Loading Response
LS: Left cross-Slope
MCC: Matthew’s Correlation Coefficient
M-SCKAFO: Microprocessor-controlled Stance-Control Knee-Ankle-Foot Orthoses
MSD: Mean Standard Deviation
PO: Push-Off
RS: Right cross-Slope
SCKAFO: Stance-Control Knee-Ankle-Foot Orthoses
SP: Self-Paced
TN: True Negative
TP: True Positive
TSVC: Transition Sequence Verification and Correction
TSw: Terminal Swing
US: Up Slope
WEKA: Waikato Environment for Knowledge and Analysis
